# Placental inflammation is associated with rural and remote residence in the Northern Territory, Australia: a cross-sectional study

**DOI:** 10.1186/s12884-015-0458-7

**Published:** 2015-02-14

**Authors:** Cecelia M O’Brien, Susan Arbuckle, Sujatha Thomas, Jurgen Rode, Robin Turner, Heather E Jeffery

**Affiliations:** Department of Obstetrics and Gynaecology, Royal Darwin Hospital, Tiwi, Northern Territory Australia; Department of Anatomical Pathology, The Children’s Hospital, Westmead, New South Wales Australia; Department of Anatomical Pathology, Royal Darwin Hospital, Tiwi, Northern Territory Australia; School of Public Health, University of Sydney, Sydney, New South Wales Australia; Discipline of Obstetrics and Gynaecology, The University of Adelaide, Women’s and Children’s Hospital, North Adelaide, 5006 South Australia Australia

**Keywords:** Placenta, Indigenous women, Pregnancy, Remote, Residence, Genitourinary infections, Anaemia, Smoking, Histological chorioamnionitis, Fetal inflammatory response

## Abstract

**Background:**

The Northern Territory has the highest rates of perinatal morbidity and mortality in Australia. Placental histopathology has not been studied in this high-risk group of women.

**Methods:**

This is the first study to detail the placental pathology in Indigenous women and to compare the findings with non-Indigenous women in the Northern Territory. There were a total of 269 deliveries during a three-month period from the 27^th^ of June to the 27^th^ of August 2009. Seventy-one (71%) percent of all placentas were examined macroscopically, sectioned then reviewed by a Perinatal Pathologist, blinded to the maternal history and outcomes.

**Results:**

Indigenous women were found to have higher rates of histologically confirmed chorioamnionitis and or a fetal inflammatory response compared with non-Indigenous women (46% versus 26%; OR 2.4, 95% CI 1.3-4.5). In contrast, non-Indigenous women were twice as likely to show vascular related pathology (31% versus 14%; OR 2.77, 95% CI 1.3-5.9). Indigenous women had significantly higher rates of potentially modifiable risk factors for placental inflammation including genitourinary infections, anaemia and smoking. After adjusting for confounders, histological chorioamnionitis and fetal inflammatory response was significantly associated with rural or remote residence (Adjusted OR 2.5, 95% CI 1.08 – 5.8).

**Conclusion:**

This study has revealed a complex aetiology underlying a high prevalence of placental inflammation in the Northern Territory. Placental inflammation is associated with rural and remote residence, which may represent greater impact of systemic disadvantage, particularly affecting Indigenous women in the Northern Territory.

## Background

The Northern Territory (NT) has the highest rates of perinatal morbidity and mortality in Australia. Women who identify as Aboriginal or Torres Strait Islander on average have two to three times the rate of preterm delivery, low birth weight and fetal death, along with increased rates of maternal morbidity and mortality in comparison to non-Indigenous women [1-3].

Epidemiological studies have shown high rates of smoking [4], remote residence [5] and sexually transmitted infections [6] in Indigenous women are associated with poor perinatal outcomes. However, little is known about the underlying pathology contributing to the high rates of perinatal morbidity and mortality in the Northern Territory.

Firstly, high rates of smoking have been reported in Indigenous women living in urban and rural and remote areas within the Northern Territory (NT) [1-3,7]. In the most recent Mothers and Babies report from the NT [7], 50.6% of Indigenous women smoked during the first 20 weeks and 47.7% continued to smoke after 20 weeks of pregnancy. Wills and Coory found the number of Indigenous mothers who smoked was three times the rate of non-Indigenous mothers. Indigenous women who smoked had a higher rate of low birth weight infants compared to non-Indigenous smokers (5.3% versus 3.7%). The rate of preterm delivery was marginally increased from 6.1% (in non-Indigenous non-smokers) to 8.3% in Indigenous women who smoked [4].

Secondly, there is strong evidence to support the role of infection and inflammation in the aetiology of premature rupture of membranes [8], preterm delivery [9] and stillbirth [10]. Interestingly, high rates of sexually transmitted diseases (20.2%) in Indigenous women have been associated with low birth weight and perinatal mortality [6]. Genitourinary infections are modifiable risk factors that can lead to ascending infection and histological chorioamnionitis. This stimulates the inflammatory cascade leading to premature rupture of membranes and preterm delivery [8].

Histological chorioamnionitis is an important and frequent cause of preterm birth, low birth weight and fetal death. A population based study in Australia by Gordon and associates in 2011 found that out of 952 fetal deaths, histological chorioamnionitis was found in 22.6% of cases [10,11]. In a large cohort of preterm infants born between 20 and 34 weeks in Australia, histological chorioamnionitis was found in 31% of the total 3928 neonates included in this study [9].

Since the establishment of this causal pathway, the evidence for screening, detecting and treating genitourinary infections in the prevention of preterm delivery is emerging. Kiss and associates reported one of the first randomised controlled trials investigating the role of a simple screening program for asymptomatic vaginal infections in 4155 women in the Vienna in 2001 [12]. Since then, there have been further trials and now systematic reviews and meta-analyses that support the use of antibiotic treatment of asymptomatic bacteruria [13] and bacterial vaginosis [14]. A recent systematic review and meta-analysis has shown a reduction in preterm birth with the detection and treatment of intermediate flora or bacterial vaginosis, (two trials including 894 women, RR 0.54, 95% CI [0.35 – 0.84]) [14,15]. A second systematic review of asymptomatic genital infections in the prevention or reduction of preterm delivery included one eligible trial by Kiss and associates (4155 women, RR 0.48, 95% CI [0.34 – 66]) [12,15]. In both meta-analyses, preterm delivery rates have been reduced by 40 to 50%. Costs, antibiotic adverse effects and the risk of antibiotic resistance, remain concerns with ongoing debate regarding the most effective type and route of administration (vaginal or oral) [14,15].

Thirdly, a review of the antenatal service in the Northern Territory found that 27% of Indigenous women were anaemic in pregnancy [16], and there is evidence to show that folate and iron deficiencies affect humoral and cell mediated immunity [16-19]. In addition, there have been reports of associations between iron and folate deficiencies with preterm delivery and low birth weight infants [20,21].

Lastly, 25% of all women who delivered in the Northern Territory in 2011, resided in rural or remote areas and 89% of these women identified as Indigenous [3]. This challenges the delivery of adequate and timely maternity care, education, appropriate nutrition and general healthcare. Lower socioeconomic status in people residing in remote areas of the Northern Territory, has been shown to influence food choices, contributing to energy dense, nutrient poor diets [22]. The most recent epidemiological study by Steenkamp and associates in 2012 [5], describes a retrospective review of maternal and perinatal outcomes, including a multivariate analysis using factors such as Indigenous status, remoteness and region within the Northern Territory. This study showed ‘remote-dwelling’ Indigenous mothers had more antenatal risk factors and poorer outcomes compared with non-Indigenous women. This included younger age at delivery, less pain relief in labour, higher caesarean section rates and longer hospital stays for vaginal births, poorer neonatal outcomes including higher rates of low birth weight and preterm births. Remoteness was found to be an independent risk factor in this study by Steenkamp [5].

Despite the high rates of perinatal morbidity and mortality, there have been no studies examining the placentae from women residing in the Northern Territory or generally in women who identify as Aboriginal or Torres Strait Islander within Australia.

We hypothesise that placental inflammation may contribute to the perinatal morbidity and mortality in the Northern Territory. The aim of this study was to undertake macroscopic and histological examination of placentas from a cohort of women delivering at the Royal Darwin Hospital to determine the overall prevalence of placental pathology in women delivering at the Royal Darwin Hospital. The second aim was to compare placental pathology in Indigenous and non-Indigenous women. The third aim was to correlate placental pathology with maternal demographics, obstetric and neonatal outcomes.

## Methods

This cross-sectional, descriptive study examined placentas from a cohort of women who delivered at the Royal Darwin Hospital during the 27^th^ of June and the 27^th^ of August 2009. During this period, there were a total of 270 deliveries and 192 (71%) placentas were examined as part of the study. Ethics committee review and approval was obtained from the Human Research Ethics Committee of the Northern Territory Department of Health and Families and Menzies School of Health Research with approval number 08/86.

The inclusion criteria included all consecutive singleton deliveries, greater than 20 weeks gestation or greater than 500 grams who delivered at Royal Darwin Hospital during a three month period from June to August 2009. The only exclusion criterion was multiple pregnancies.

Women were predominately recruited in the postpartum period. Informed written consent was obtained after their consideration of the information leaflet and discussion with the midwife, researcher, interpreter or Indigenous Liaison Officer. After the delivery, verbal consent for participation was given to the midwife caring for the woman and the placenta was placed in a special dedicated fridge in labour ward. Within 24 hours of delivery, written and informed consent was obtained by the primary researcher, and the placentas were placed in buffered formalin in a sealed plastic container and transported to the Department of Anatomical Pathology. Twenty percent of women were recruited in the antenatal clinic during their second and third trimesters visits.

A pilot study examining 24 placentas was conducted prior to the commencement of the study to ensure quality assurance of the macroscopic examination of the placenta. For each placenta, the primary researcher (advanced obstetric trainee) and the senior pathology registrar performed the macroscopic examination together. All histopathology slides were then reviewed by the Head of Anatomical Pathology to ensure that the sections were standardised. The placentas were examined, sectioned and prepared according to the College of American Pathologists (CAP) guidelines [23], based on the Benirschke and Kaufmann method [24].

A Perinatal Pathologist at the Children’s Hospital at Westmead (SA) examined 174 (90.6%) placentas for this study during 2010 and 2011. SA was blinded to all clinical information including the gestation, ethnicity, and obstetric and neonatal outcome.

There were 18 placentas (9.4%) examined during the postpartum period for a clinical indication to assist in the management of the newborn and future pregnancies. These placentas were examined using the same method as described above in the Department of Anatomical Pathology at the Royal Darwin Hospital by the Pathology Registrar and reviewed by the Head Anatomical Pathologist (JR).

The definitions of placental inflammation were based on the criteria from Redline and colleagues [25]. Histological chorioamnionitis is defined as the infiltration of neutrophils into the amnion and/or chorion in response to a bacterial infection. The maternal inflammatory response starts at the chorionic plate and at the junction between the decidua and chorion in the first 12 hours. Within 24 hours, there is migration and spread into the amnion, enabling the organisms to then enter the amniotic cavity. The fetal inflammatory response occurs later with migration of fetal neutrophils beginning in the umbilical vein or chorionic vessels, migrating over time to the umbilical arteries and into the Wharton’s jelly. It is usually clinically silent and is diagnosed by a pathologist after delivery. Funisitis was defined as the focal infiltration of neutrophils at the umbilical cord surface and in the Wharton’s jelly.

The definition of a **high-risk pregnancy** included women with significant co-morbidities (essential hypertension, rheumatic heart disease, pre-existing diabetes, thrombophilia), past or current pregnancy complications (previous preterm delivery, growth restriction, fetal death, previous early onset pre-eclampsia, gestational diabetes). This signified the need for high-risk antenatal care by an Obstetric team throughout the pregnancy. The definition of **low risk pregnancy** included women with no co-morbidities and no past or current pregnancy complications. This group had predominately midwifery care throughout the pregnancy.

**Anaemia** was defined as a Haemoglobin level of less than 110 g/dL.

**Rural, Remote and Metropolitan Area (RRMA) classification:** Postcodes and place of residence was collected as part of the demographic data for the study. Using the RRMA classifications, each participant was individually classified as metropolitan, rural or remote. Metropolitan zone is defined as any capital city or centre with an urban population greater than or equal to 100,000. Rural zone was defined as small or large rural centre with population ranging between 10,000 and 99,999. Remote zone included remote centres, defined as greater than 5,000 but less than 10,000 and other remote areas with a population less than 5,000 [26].

Birth weight, head circumference and length **percentiles** were obtained from the on-line calculator based NSW data from Doctor Philip Beeby from Royal Prince Alfred Hospital in Sydney [27]. The primary reason for the use of NSW data in preference to the Australian National data was the access to online calculation generating a precise percentile calculation rather than an estimate or a range.

The **sample size** was calculated based on the baseline prevalence of chorioamnionitis in term pregnancies in a large cohort study by Russell and associates from Royal Prince Alfred Hospital in 1979 [28]. Our assumption was that there would be increased rates of chorioamnionitis in our study compared with the population of women in Sydney. Using a power of 80% to detect a difference in the rate of chorioamnionitis of 25% (5% versus 30%), assuming a significance level of p < 0.05. The minimal sample size calculated was 86 (43 in each group). Using a proportion of 2:1 (non-Indigenous compared with Indigenous births), the sample size was 102 (68 placentas in non-Indigenous women and 34 in Indigenous women).

### Statistical analysis

The demographic data in this study was analysed using the t-statistic for continuous variables and the chi-squared statistic for dichotomous and nominal variables. Multivariate analysis was undertaken to assess the impact of ethnicity on placental inflammation after adjusting for perinatal factors. These factors included maternal age, gestational age, smoking, prolonged ruptured membranes, location (rural/remote versus metropolitan), genitourinary infection and anaemia. There were five categories for gestation in completed weeks: 20 – 36 weeks, 37 weeks, 38 weeks, 39 weeks and 40 and above weeks. Age was used as a continuous variable within the model. The final model was developed using stepwise logistic regression. Odds ratio and adjusted odds ratio with 95% confidence intervals were reported. All P-values were two sided at a significance level of P < 0.05. Analyses were conducted in SPSS version 19.0.0 [29] and R Program [30].

## Results

**Figure 1:**
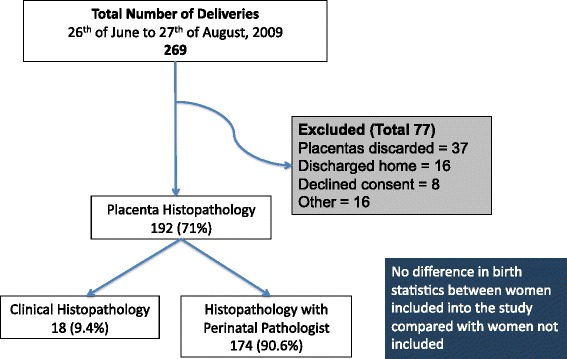
**Flow chart for this cross-sectional study.** There were 269 singleton deliveries during the study period between the 27^th^ of June and the 27^th^ of August 2009. Seventy-seven placentas were not included for reasons including declined consent (8), discharge home prior to consent (16) or discarding of placentas post delivery (37) and other reasons (16). A total of 192 placentas were included in this study, representing 71% of the entire cohort of deliveries. There were no statistically significant differences when comparing women included with those excluded from the study. A perinatal pathologist, blinded to outcome, gestation and ethnicity, reviewed over 90% of the placentas. Nine percent were examined for clinical reasons in the Department of Anatomical Pathology, Royal Darwin Hospital.

During the study period between the 26^th^ of June and the 27^th^ of August 2009, there were 269 women who delivered 269 singleton newborns. One hundred and ninety-two mothers and newborns were included in the cross-sectional study, representing 71% of all deliveries in this time period. There were no significant differences in the birth statistics of women included in the study in comparison to women who were not included in the study as shown in Figure 1. This included age, ethnicity, parity, location of usual residence, mode of delivery, birth weight, gestation and sex of the infant. Figure 1 shows the flow chart for the Placental study. Of the 192 women included in the study, 18 (9.4%) of placentas were sent for histopathology at the Royal Darwin Hospital for clinical reasons such as perinatal mortality, fetal distress, preterm delivery, low apgars and clinical signs of chorioamnionitis.

All baseline characteristics for Indigenous and non-Indigenous women included in this study are shown below in Table 1. In this study, 59 (30.7%) out of the 192 placentas did not show any pathology. An inflammatory response in the amnion or chorion or umbilical cord was found in a total of 65 placentas (33.9%). Fourty-six placentas (24%) showed vascular pathology as the primary finding. Confirmed villitis and related pathology was found in 13 (6.8%) cases.

**Table 1:** **Baseline characteristics in the cross-sectional study**

	**Indigenous n (%)**	**Non-indigenous n (%)**	**Missing n (%)**	**Significance**
	**N = 79**	**N = 113**		
Maternal Age (years) – *median (IQR)*	27 (22–32)	26 (22–32)	0	P = 0.43
Parity – *median (IQR)*	2 (1 – 3)	1 (0 – 2)	1	P < 0.0001**
***Area of residence (RRMA)***				
Metropolitan	18 (22.8)	97 (85.8)	0	Analysis of
Rural	4 (5.1)	13 (11.5)	0	Metro vs Rural and Remote P < 0.0001**
Remote	57 (72.2)	3 (2.7)	0	
Smoking	37 (46.8)	35 (31)	30 (16)	P = 0.03*
BMI < 20 (kg/m^2^)	3 (3.8)	1 (0.9)	83 (43)	P = 0.04*
BMI > 30 (kg/m^2^)	10 (26.5)	30 (12.6)	83 (43)	P = 0.02*
***Pregnancy related***				
Antenatal Care (less than 4 visits)	11 (13.9)	2 (1.8)	1 (0.5)	P = 0.001*
High risk classification	46 (58.2)	25 (22)	0	P < 0.0001**
Gestational Diabetes	13 (16.5)	4 (3.5)	22 (11.5)	P = 0.002*
Hypertension	11 (13.9)	21 (18.6)	8 (4.2)	P = 0.39
Anaemia	13 (16.5)	5 (4.4)	0	P = 0.005*
Candida culture positivity	24 (30.4)	4 (3.5)	0	P < 0.0001**
Urinary tract infection	15 (19)	7 (6.2)	0	P = 0.006*
GBS positivity (3^rd^ Trimester)	13 (24.5)	21 (21.4)	41 (21.4)	P = 0.6
Sexually transmitted disease (1^st^ Trimester)	13 (16.5)	1 (0.9)	97	P < 0.0001**
***Mode of Delivery***			0	Analysis of Vaginal vs LSCS P = 0.03*
Spontaneous Vaginal Delivery	48 (61)	80 (71)		
Instrumental Vaginal Delivery	4 (5.1)	10 (8.8)		
Emergency LSCS	18 (23)	15 (13.3)		
Elective LSCS	9 (11.4)	8 (7)		
***Neonatal Outcomes***				
*Gestation (weeks) – median*	39 wks	39.5 wks	0	P = 0.005*
Preterm deliveries (%) < 34 wks	8 (10.1)	3 (2.7)		P = 0.03*
<37 wks	12 (15.2)	8 (7.1)		P = 0.07
*Birth weight (grams) – median*	3215 grams	3365 grams	0	P = 0.01*
Low birth weight < 2500 gms (%)	15 (19.0)	10 (8.8)	0	P = 0.04*
Small for Gestational Age (<10^th^)	12 (15.2)	10 (8.8)	13 (6.8)	P = 0.18
Large for Gestational Age (>90^th^)	10 (12.6)	9 (7.9)	13 (6.8)	P = 0.27
Admission into the NICU	27 (34.2)	21 (18.6)	6 (3.1)	P = 0.02*

NB: Denominator for each column = N, numerator = n, % is the percentage of the numerator/denominator for each column. There is missing data for some of these categories and the statistical analysis included all missing data in the denominator.

*P = 0.05 – 0.0001, **P < 0.0001.

**Figure 2:**
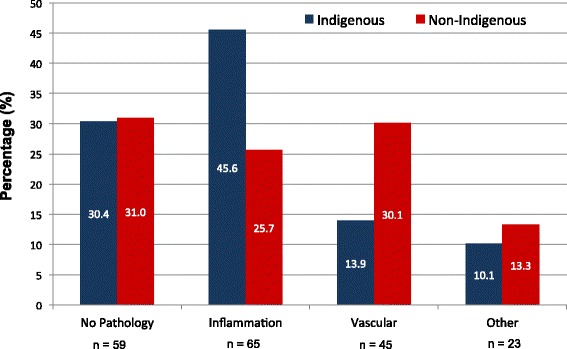
**Prevalence of primary placental pathology according to ethnicity (N = 192).** The X-axis depicts the 4 types of primary placental pathology with the total number of women in each group below. The Y-axis is the percentage of cases in each pathology group based on ethnicity with the denominator being the total number of women in each ethnicity group (Indigenous = 79, Non-Indigenous = 113). The percentage of cases in each pathology group is shown inside the columns. There was a significant difference in the prevalence of placental inflammation in Indigenous women compared with non-Indigenous (45.6% vs 25.7%). In contrast, non-Indigenous women had a higher prevalence of vascular pathology compared with Indigenous women (30.1% vs 13.9%). There were no differences in the ‘No Pathology’ and ‘Other’ pathology comparing Indigenous women to non-Indigenous women.

There was a significant association between placental inflammation and ethnicity (χ^2^ = 8.5, df = 1, p = 0.004) as shown in Figure 2. Thirty-six Indigenous women (45.6%) were found to have confirmed inflammation on placental examination in comparison to 29 of non-Indigenous women (25.7%). Indigenous women were twice as likely to have histological chorioamnionitis and or a fetal inflammatory response (OR 2.43 with 95% CI 1.3 to 4.5).

There was a significant association between the vascular pathology and ethnicity (χ^2^ = 7.42, df = 1, p = 0.006). A total of 35 non-Indigenous women (31%) had vascular primary pathology compared with 11 Indigenous women (13.9%). The odds ratio was 2.78 (95% CI 1.3 and 5.9).

The overall prevalence of histological chorioamnionitis was 33.9% in this cross-sectional study of women delivering at the Royal Darwin Hospital. The term placental inflammation rate was 32.6% (56 out of a total of 172 term infants). The overall prevalence of chorioamnionitis in women residing in urban Darwin was 24.3% (n = 115) and in women classified as low risk, the prevalence was 33.1% (n = 121). When these three factors were combined, 88 women were term, low risk and lived in urban Darwin. The baseline rate of histological chorioamnionitis with or without a fetal inflammatory response in this group of women was 25.6%.

**Figure 3:**
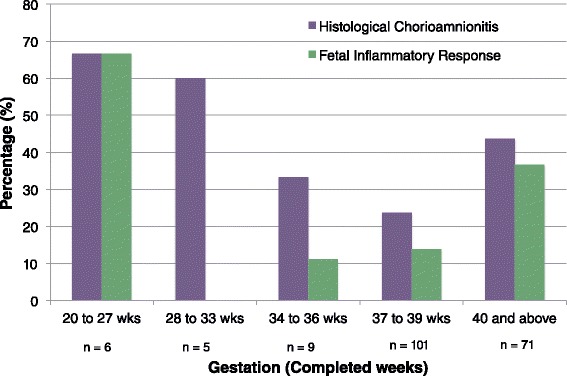
**Prevalence of histological Inflammation according to gestation and ethnicity.** This graph demonstrates a bimodal relationship with gestation and placental inflammation. At the extremes of gestation, placental inflammation less than 28 weeks is over 60% and for women delivering at 40 weeks and beyond, the prevalence is approximately 40%. Between 37 and 39 completed weeks, the prevalence of histological chorioamnionitis reaches the minimal value of 24%. The fetal inflammatory response including funsitis is more pronounced at the extremes of gestation, and was absent in women delivering between 28 and 33 completed weeks.

The preterm inflammation rate was 45% (9 out of 20 preterm infants). There was no association between the presence of chorioamnionitis and all preterm delivery (χ^2^ = 1.802, df = 1, P = 0.18) or spontaneous preterm delivery (χ^2^ = 3.03, df = 1, P = 0.08). Figure 3 illustrates higher rates of histological chorioamnionitis and fetal inflammatory response at the extremes of gestation, below 28 weeks and 40 weeks and above.

Table 2 shows the results of the univariate analysis of histological chorioamnionitis with or without a fetal inflammatory response and the association with multiple variables including potential risk factors and pregnancy outcomes.

**Table 2:** **Univariate analysis of the perinatal risk factors associated with placental inflammation**

**Perinatal Factors**	**Total**	**Missing data**	**Placental inflammation**	**P value**
	**N = 192**		**N = 65**	
	**n (%)**		**n (%)**	
***Maternal Age (years)***				
Median (inter-quartile range)		0	24 (21 – 31)	P = 0.04*
			27 (23 – 32)	
			No inflammation	
***Parity***				P = 0.07
*Nulliparous*	60 (31.4)	0		26 (43.3)
*Multiparous*	131 (68.6)		39 (29.8)	
***Residence***				P < 0.0001** P = 0.001*
*Metropolitan*	115 (59.9)	0	28 (24.3)	
*Rural*	17 (8.9)	0	6 (35.3)	
*Remote*	60 (31.3)	0	**31 (51.7)**	
*Metropolitan and Rural versus Remote*	132 (68.8)	0	34 (25.8)	
*Rural and Remote vs Metropolitan*	77 (40)	0	37 (56.9)	
***Ethnicity***				P = 0.004*
*Indigenous*	79	0	36 (45.6)	
*Non-Indigenous*	113	0	29 (25.7)	
***High risk***				P = 0.8
*Yes*	71 (37)	0	25 (35.2)	
*No*	121 (63)	0	40 (33.1)	
***Less than 4 antenatal visits***				P = 0.8
*Yes*	13 (6.8)	0	4 (30.8)	
*No*	178 (92.7)	1	60 (34.3)	
***Anaemia***				P = 0.04*
*Yes*	18 (9.4)	0	10 (55.6)	
*No*	174 (90.6)		55 (31.6)	
***BMI ≤ 20 kg/m*** ^***2***^				P = 0.04*
*Yes*	4 (2.1)	83 (43.2)	3 (75)	
*No*	105 (54.7)		29 (27.6)	
***BMI ≥ 30 kg/m*** ^***2***^				P = 0.23
*Yes*	40 (20.8)	83 (43.2)	9 (22.5)	
*No*	69 (35.9)		23 (33.3)	
***Smoking***				P = 0.076
*Yes*	72 (37.5)	30 (15.6)	30 (41.7)	
*No*	90 (46.9)		35 (29.2)	
***1*** ^***st***^ ***Trimester Genitourinary positive culture***				P = 0.057
Yes	57 (29.7)	97 (50.5)	25 (38.5)	
No	38 (19.8)		40 (29.6)	
***Candida culture positivity***				P = 0.05*
Yes	28 (14.6)	0	14 (50)	
No	164 (85.4)		51 (31)	
***Urinary Tract Infection***				P = 0.09
*Yes*	22 (11.5)	0	11 (50)	
*No*	170 (88.5)		54 (31.8)	
***Sexually transmitted disease***				P = 0.9
*Yes*	14 (7.3)	97 (50.5)	5 (35.7)	
*No*	81 (42.2)		60 (33.7)	
***Any Genitourinary culture positivity***				P = 0.5
*Yes*	70 (40.9)	21 (10.9)	26 (37.1)	
*No*	101 (59.1)		30 (29.7)	
***GBS positivity***				P = 0.6
*Yes*	34 (22.5)	41 (21.4)	13 (38.2)	
*No*	117(77.5)		52 (32.9)	
***Rupture of membranes > 48 hrs***				P = 0.004*
*Yes*	9 (4.7)	0	7 (77.8)	
*No*	183 (95.3)	0	58 (31.7)	

Denominator for each column = N, numerator = n, % is the percentage of the numerator/denominator for each column.

Missing data was not excluded in the denominator. *p = 0.05 – 0.001, **p < 0.001.

The fetal inflammatory response was present in 46 (70.8%) out of the 65 cases showing placental inflammation. The more severe response defined as funisitis, occurred in 27 of the total 46 cases with a confirmed fetal inflammatory response (48.7%). There was a significant increase in the rate of funisitis in Indigenous women (20.3%) compared with non-Indigenous women (9.7%) (χ^2^ = 8.14, p = 0.04). The odds ratio was 2.08 (95% CI 1.02 - 4.24), indicating that Indigenous women have twice the rate of funisitis compared with non-Indigenous women.

There were a total of 71 high-risk pregnancies and 121 low risk pregnancies in this study. There were no differences in placental inflammation when women classified as high risk (35.2%) were compared with women who were classified as low risk (33.1%) (χ2 = 0.93, p = 0.7). When all high-risk pregnancies were excluded from the analysis, a reanalysis was performed of low risk pregnancies alone. There were significant differences in placental inflammation for Indigenous women (51.5%) when compared with non-Indigenous women (26.1%) (χ^2^ = 6.985, P = 0.008). The odds ratio was 3.0 (95% CI 1.31 – 6.9) indicating in low risk pregnancies, Indigenous women were three times more likely to show placental inflammation compared with non-Indigenous women.

Women who resided in Darwin and outer surrounding suburbs were included in a specific analysis (n = 115), with exclusion of women living rurally or remotely (n = 77). There were a total of 28 cases of placental inflammation with an overall prevalence of placental inflammation to be 24.3%. There were no significant differences in the prevalence of placental inflammation in Indigenous women (22.2%) compared to non-Indigenous women (24.7%) (χ^2^ = 0.52, P = 0.89), whose place of residence and primary catchment area was Darwin and surrounding suburbs.

There were a total of 60 (31.3%) women who were classified as living remotely using the RRMA classification. There were 132 women who were non-remote (68.8%). Indigenous women more likely to live remotely (57 of 79, 72.2%) and non-Indigenous women were more likely to live in non-remote areas of the Northern Territory (110 of 113, 97.3%). Placental inflammation occurred in 51.7% women who lively remotely compared with 25.8% who lived in non-remote areas (χ^2^ = 12.37, P < 0.0001). The odds ratio was 3.08 (95% CI 1.6 – 5.8), indicating that women who lived remotely had three times the prevalence of placental inflammation compared with women living in metropolitan or rural areas.

The multivariate analysis is shown in Table 3 and the model was adjusted for confounders including gestation, maternal age, premature rupture of membranes greater than 48 hours and rural or remote residence. There was no difference in placental inflammation comparing Indigenous to non-Indigenous women after adjusting for confounders (Adjusted OR 1.62 [0.68 – 3.84]). Interestingly, women who lived in rural and remote residences had a 2.5 times increased risk of developing placental inflammation (Adjusted OR 2.5 [1.08 – 5.84]).

**Table 3:** **Multivariate analysis of Placental inflammation**

**Regression model**	**Unadjusted odds ratio (95% CI)**	**Adjusted odds ratio (95% CI)**	**Significance**
Gestation	0.96 (0.88 – 1.04)	0.17 (0.06 – 0.55)	P = 0.002
Maternal age	0.95 (0.9 – 0.997)	0.95 (0.89 – 0.998)	P = 0.04
Prolonged rupture of membranes > 48 hours	7.5 (1.52 – 5.3)	10.5 (1.73 – 63.8)	P = 0.009
Residence			P = 0.03
*Rural and remote versus Metropolitan*	2.9 (1.55 – 5.3)	2.5 (1.08 – 5.84)	
Ethnicity			P = 0.28
*Indigenous vs Non-Indigenous*	2.4 (1.3 – 4.5)	1.62 (0.68 – 3.84)	

Multivariate analysis was undertaken to assess the impact of ethnicity on placental inflammation after adjusting for perinatal factors. These factors included maternal age, gestational age, smoking, prolonged ruptured membranes, location (rural/remote versus metropolitan), genitourinary infection and anaemia. There were five categories for gestation in completed weeks; 20 – 36 weeks, 37 weeks, 38 weeks, 39 weeks and 40 and above weeks. Age was used as a continuous variable within the model. The final model was developed using stepwise logistic regression.

## Discussion

This is the first study to comprehensively describe placental pathology from women delivering at the Royal Darwin Hospital, a tertiary referral centre in the Top End of the Northern Territory.

In this study, 32.6% of placentas from women delivering at term showed inflammation of the amnion, chorion or umbilical cord. A detailed analysis was performed to determine the baseline prevalence of placental inflammation in low risk, term women in this study who lived in the primary catchment area for Royal Darwin Hospital. The overall prevalence of histological chorioamnionitis in women residing in urban Darwin was 24.3% (n = 115); in women delivering at term was 32.6% (n = 172) and in low risk women was 33.1% (n = 121). Combining the three variables resulted in 88 women who were term, low risk and lived in Darwin and the baseline prevalence of placental inflammation was 25.6%.

The most recent study reporting the prevalence of chorioamnionitis in term pregnancies, examined 1012 placentas as part of a population based Auckland Birth weight Collaborative (ABC) study in New Zealand, 2010. The overall prevalence of histological chorioamnionitis was found to be 14.3%, with maternal inflammation alone contributing to over 50%, both maternal and fetal inflammation found in 45.5% and fetal inflammation alone in 2.8% [31]. In comparison to our current study, we found an increased prevalence of HCA and fetal inflammatory response (66%), with less HCA alone (29%) and a small increase in fetal inflammation alone (4.6%). In the ABC study, there were higher incidences of amnionitis in women who identified as Chinese (25.8%, n = 93), Maori (21.5%, n = 65) and Pacific Islanders (16.4%, n = 122) compared with European (12.2%, n = 555) women in the multivariate analyses. Interestingly, ethnic differences in the ABC study persisted after adjusting for confounders [31].

The prevalence of histological chorioamnionitis in our study was 1.5 times the prevalence reported in the recent ABC study in New Zealand [31] and over 5 times the rate reported in a cohort study in RPA in 1979 [28]. In the RPA study, the rate of acute chorioamnionitis was reported to be 3.8% for 37 to 40 weeks and 5.1% for 41 to 44 weeks gestation [28]. Comparatively, international data from a 16-year retrospective study of placental histopathology in Hungary revealed subclinical acute chorioamnionitis occurred in 5.1% of term pregnancies compared with 30% in preterm pregnancies [32].

Differences in the prevalence of histological chorioamnionitis could be due to the wide range of sampling techniques and diagnostic criteria. The ABC study [31] only sampled the chorionic vessels and placental parenchyma and did not perform a membrane roll to assess the chorion and amnion separately or sample the umbilical cord to assess the extent of the fetal inflammatory response. The RPA study [28] used similar sampling techniques including a membrane roll but used one sample of the umbilical cord. In our study, we performed a membrane roll and obtained 2 samples of the umbilical cord, one from the fetal end and one from the chorionic end.

Potential mechanisms underlying the higher prevalence of histological chorioamnionitis in this study include ascending infection, immune function, malnutrition and systemic disadvantage. There were trends towards significance for 1^st^ trimester genitourinary infections and significant differences for candida and placental inflammation in our study (Table 2). There was a moderate quantity of missing data for 1^st^ trimester genitourinary testing and this was predominately in the non-Indigenous women from the primary catchment area. However, the majority of women had a 3^rd^ trimester low vaginal swab for group B streptococcus screening (Table 2). Interestingly, Indigenous women had a significantly higher rate of culture positive candidiasis (30.4% vs 3.5%) and urinary tract infections (19% versus 6.2%) compared with non-Indigenous women.

A recent Cochrane review has reported that the treatment of women with abnormal vaginal flora reduced the risk of preterm delivery by 47% (2 trials, n = 894, RR 0.53 [0.34-0.84]) [14]. In our study, the prevalence of candidiasis was 14.6%, and was associated with placental inflammation. This rate is comparable to the large randomised controlled trial by Kiss and colleagues in Vienna in 2004, where a simple screening program for abnormal vaginal flora was shown to reduce the rate of preterm delivery by 50% [33]. Ongoing studies, examining the treatment of candida and the effect on preterm delivery rates, are being conducted [33]. A well-designed, randomised controlled trial would be the next step to determine if screening and treatment of genitourinary infections in each trimester could reduce chronic or recurrent genitourinary infection, powered to assess clinical outcomes such as preterm delivery and include histopathology to determine underlying placental inflammation.

Poor nutrition and smoking have been postulated to cause low folate levels and the former iron deficiency that may underlie the high rates of anaemia in Aboriginal and Torres Strait Islanders [34]. In our study, anaemia was reported to be present in 16.5% of Aboriginal and 4.4% of non-Aboriginal pregnant women. Anaemia was also significantly associated with the presence of placental inflammation. Naeye and colleagues found that in Ethiopian women, amniotic fluid infection was higher in those who were malnourished and impoverished [35]. Host defense factors that normally exist to prevent infection were reduced in malnourished pregnant women [36]. In the developing world, poor nutrition and sub-optimal antenatal care is associated with a higher incidence of infection [37]. This study did not test for micronutrient deficiencies or dietary factors in pregnancy, but further study in this area may help to identify potentially reversible factors that may impact the onset and chronicity of placental inflammation.

Remoteness could be a proxy for other factors such as socioeconomic disadvantage including education, housing, employment and income [5]. The multivariate analysis showed ethnicity is not associated with placental inflammation after adjusting for confounders. Interestingly, rural and remote residence remained significant risk factors. The multivariate analysis incorporated tests for co-linearity with no significant effect between ethnicity and residency. Graham and colleagues postulate that ‘systemic disadvantage’ rather than suboptimal care in pregnancy is likely to be the cause of inequalities seen in perinatal outcomes for Indigenous women living in rural and remote areas in Australia [38]. This was supported in our study were there were no associations with suboptimal antenatal care and placental inflammation.

The relationship with gestation is an interesting finding. From the literature, there is an inverse and linear relationship between chorioamnionitis and preterm gestation [9]. Russell and associates found high rates of acute chorioamnionitis in 94% cases between 21-24 weeks, 40% in 25 – 28 weeks, 35% between 29 – 32 weeks and 10% in 33 – 36 weeks, 3.8% at term and 5.1% over 40 weeks [28]. There have been two recent studies examining cases of fetal death that have shown a bimodal relationship with gestation [10,11]. In 2007, Lahra and colleagues reported a bimodal relationship with gestation in a 15-year hospital cohort study examining placental inflammation and fetal death [11]. The rate of chorioamnionitis in extreme premature and post mature gestations was over 50% with an overall rate of 36.9%. A population cohort study of fetal deaths in Australia between 2002 and 2004 found the incidence of histological chorioamnionitis to be 22.6% and fetal inflammatory response was 10.1%, most pronounced after 40 weeks gestation. Both studies found that the fetal inflammatory response was associated with spontaneous labour and the lack of this response was associated with an unexplained fetal death [10,11]. There is good evidence to support that infection and inflammatory changes contribute to onset of labour in preterm deliveries [8]. There remains uncertainty concerning histological chorioamnionitis and fetal inflammatory response during the term and post-term period. Hypotheses include a reaction to meconium [39,40], prolonged rupture of membranes [41], coitus [42], progressive cervical effacement [43], cervical dilatation and multiple vaginal examinations in labour [44]. At least two studies have found that meconium stained amniotic fluid and neonatal morbidity is associated with placental inflammation [39,40]. There is some evidence to support that the hypothesis that meconium may predispose and increase the incidence of infection and related histological placental inflammation [10,11,40]. In our study, there was no difference in the presence of meconium and placental inflammation (10.4% compared with 14.2% with no inflammation) nor were there differences in neonatal outcomes (Table 4).

**Table 4:** **Perinatal outcomes relating to the presence of placental inflammation**

	**Total**	**No inflammation**	**Placental inflammation**	**P value**
	**N = 192**	**N = 127**	**N = 65**	
	**n (%)**	**n (%)**	**n (%)**	
**Intrapartum**				
*Clinical syndrome*	7 (3.6)	1 (0.8)	6 (9.2)	P = 0.003*
*Intrapartum fever*	9 (4.7)	2 (1.6)	7(10.8)	P = 0.004*
*Fetal distress*	56 (29.2)	35 (27.6)	21 (32.3)	P = 0.5
*Meconium*	25 (13)	18 (14.2)	7 (10.8)	P = 0.5
*Caesarean Section*	50 (26)	25 (19.6)	15 (23.1)	P = 0.5
**Postnatal**				
*Endometritis*	11 (5.7)	9 (7.1)	2 (3.1)	P = 0.3
*Fever/Sepsis*	24 (12.5)	12 (9.4)	12 (18.5)	P = 0.07
**Neonatal**				
*Apgar < 4 at 5mins*	8 (4.2)	4 (3.1)	4 (6.2)	P = 0.3
*PTB < 37 weeks*	20 (10.4)	11 (8.7)	9 (13.8)	P = 0.3
*Spontaneous PTB*	17 (8.9)	9 (13.8)	8 (6.3)	P = 0.08
*SGA < 10th centile*	22 (11.5)	14 (11)	8 (12.3)	P = 0.8
*LGA > 90* ^*th*^ *centile*	16 (8.3)	14 (11)	2 (3.1)	P = 0.06
*Less than 50* ^*th*^ *centile*	100 (52.1)	63 (49.6)	37 (56.9)	P = 0.2
*Admission to NICU*	50 (26.9)	32 (25.2)	18 (27.7)	P = 0.7
*Suspected sepsis*	42 (21.9)	25 (19.7)	17 (26.2)	P = 0.3
*Confirmed sepsis*	5 (2.6)	3 (2.4)	2 (3.1)	P = 0.8
**Perinatal Mortality**	7 (3.6)	3 (2.4)	4 (6.2)	P = 0.2

An interesting finding in this cross-sectional study was the contrast in the placental pathology for Indigenous and non-Indigenous women. Non-Indigenous women showed significantly higher rates of vascular primary pathology (31%) compared with Indigenous women (14%). This included maternal under perfusion, infarction, thrombus, retroplacental haematoma and abruption. Reasons for this difference may relate to the differences in vascular related risk factors in non-Indigenous women. In this study, non-Indigenous women had significantly higher levels of obesity classified as body mass index (BMI) greater than 30 m^2^/kg (26.5% vs 12.6%) and increased rates of hypertension (essential and pregnancy related) compared to Indigenous women (18.9% versus 13.9%).

The main limitation of our study was the lack of statistical power to examine important outcomes such as preterm delivery in the setting of placental inflammation. The second limitation related to the inability to examine every placenta for the entire cohort of women delivering at Royal Darwin Hospital during this time period. Our study included 71% (192 out of 269) of all women who delivered in a 3 month time period. There were no statistically significant differences in the women who were included and those who were not included in this study. The third limitation relates to a high perinatal death noted in this study group compared with the yearly average. All fetal and neonatal deaths for this time period meet the inclusion criteria and had mandatory placental histopathology. The proportion of fetal and neonatal deaths in this three-month period is likely to represent chance rather than a significantly different group of women compared with the current population in the Top End of the Northern Territory. We also recommend further studies in Australia to test the reliability of our findings.

This study has described the complexity of placental inflammation in the Northern Territory and the cause may not be as simple as ascending infection alone. Anaemia, socioeconomic disadvantage, high rates of smoking and genitourinary infection are all likely to contribute to the prevalence of placental inflammation seen in this cohort of women in the Northern Territory. Further studies are required to understand the impact of genitourinary infections, anaemia and remote residence on rates of placental inflammation, powered to assess perinatal outcomes such as preterm delivery and low birth weight. Until we can achieve equity with housing, income, education, nutrition and access to healthcare, systemic disadvantage remains the greatest challenge for Indigenous women and their babies in the Northern Territory.

## Conclusion

This is the first study to describe the prevalence of placental pathology in the Northern Territory, Australia. The findings of this study are important in understanding the factors that contribute to the high rates of perinatal morbidity and mortality in the Northern Territory. This study found a high prevalence of subclinical, histological chorioamnionitis and fetal inflammation in both preterm and term deliveries. Potential modifiable factors were identified including anaemia and genitourinary infections such as candida. After adjusting for confounders, rural and remote residence was associated with placental inflammation, which may represent a greater impact of systemic disadvantage for women living in rural and remote Northern Territory. This study provides optimism that perinatal health may improve by correcting the socioeconomic disadvantage for women living in remote areas of the Northern Territory.

## Abbreviations

AUS: Australia
CAP: College of American Pathologists
CI: Confidence Intervals
FIR: Fetal Inflammatory Response
GUT: Genitourinary infections
HCA: Histological Chorioamnionitis
NT: Northern Territory
OR: Odds Ratio
PCR: Polymerase chain reaction
PPROM: Premature prolonged rupture of membranes
RDH: Royal Darwin Hospital
RPAH: Royal Prince Alfred Hospital
STD: Sexually transmitted disease
PTB: Preterm birth
NICU: Neonatal Intensive Care Unit
SGA: Small for Gestational Age
LGA: Large for Gestational Age
